# EDAmame: interactive exploratory data analyses with explainable models

**DOI:** 10.1093/bioinformatics/btaf340

**Published:** 2025-06-20

**Authors:** Aaron Chuah, Tim C Hewitt, Sidra A Ali, Maryam May, Tony Xu, Daniel Christiadi, Philip Y -I Choi, Elizabeth E Gardiner, T Daniel Andrews

**Affiliations:** Division of Immunology and Infectious Diseases, John Curtin School of Medical Research, Australian National University, Canberra, ACT 2601, Australia; Division of Genome Sciences and Cancer, John Curtin School of Medical Research, Australian National University, Canberra, ACT 2601, Australia; Division of Immunology and Infectious Diseases, John Curtin School of Medical Research, Australian National University, Canberra, ACT 2601, Australia; Division of Genome Sciences and Cancer, John Curtin School of Medical Research, Australian National University, Canberra, ACT 2601, Australia; Division of Immunology and Infectious Diseases, John Curtin School of Medical Research, Australian National University, Canberra, ACT 2601, Australia; Division of Immunology and Infectious Diseases, John Curtin School of Medical Research, Australian National University, Canberra, ACT 2601, Australia; Division of Immunology and Infectious Diseases, John Curtin School of Medical Research, Australian National University, Canberra, ACT 2601, Australia; Division of Genome Sciences and Cancer, John Curtin School of Medical Research, Australian National University, Canberra, ACT 2601, Australia; Canberra Health Services, Canberra Hospital, Garran, ACT 2605, Australia; Division of Genome Sciences and Cancer, John Curtin School of Medical Research, Australian National University, Canberra, ACT 2601, Australia; Division of Immunology and Infectious Diseases, John Curtin School of Medical Research, Australian National University, Canberra, ACT 2601, Australia; College of Engineering and Computer Science, Australian National University, Canberra, ACT 2601, Australia

## Abstract

**Summary:**

Complex tabular datasets comprising many diverse features can require specific expertise to interpret, posing a barrier to researchers with minimal data science experience. EDAmame is an interactive tool that simplifies initial analysis and visualization of these datasets, providing insights into data quality and feature relationships. By leveraging open-source machine learning frameworks in R, EDAmame allows researchers to perform effective exploratory data analysis without command-line or coding requirements.

**Availability and implementation:**

A limited online version can be accessed at https://edamame.org.au/ or can be downloaded from https://doi.org/10.5281/zenodo.15356492. The app is developed in R Shiny and implements tidyverse and tidymodels packages.

## 1 Introduction

Modern biological research has become a data-rich domain, yet many in the field do not yet possess the computational or data skills necessary to manipulate, visualize and analyse these data to maximally glean insights from them. Exploratory data analysis (EDA) is a critical early step in processing biological datasets in order to reveal underlying relationships that can guide research directions or further analyses (Vierheller 2014). Explorations into correlations and associations in these datasets through best-practice statistical, unsupervised and supervised machine learning (ML) techniques are integral to modern EDA.

Here, we introduce EDAmame (EDA ML-Aided Multiomics Explorer), a lightweight dataflow tool that marries the best of the classical EDA paradigm to survey data quality and underlying structure (Komorowski *et al.* 2016) with state-of-the-art ML engines. We also incorporate explainable AI (XAI), a set of methods for comprehending the reasoning behind predictions made by trained models (Minh *et al.* 2022). Simple models generated in EDAmame can be further interrogated to break-down the behaviour and influence of different features on individual predictions.

Several existing non-programmatic toolkits for statistical analyses, such as GraphPad *Prism*, Salesforce *Tableau* and *Orange* (Demsar *et al.* 2013), provide a graphical user interface to visualize and analyse datasets. Yet, they often come with such a complex variety of options that users are overwhelmed with analytical choices. These tools presume a substantial level of statistics knowledge and experience for choosing, filtering, transforming and linking components together—often needing to be done manually. Additionally, they seldom offer integrated methods of data cleaning. In biological datasets, this is of utmost importance, due to their intrinsic variability (Berger *et al.* 2013), batch effects (Leek *et al.* 2010), sampling irregularities (Metwally *et al.* 2022) and propensity for data-entry and labelling errors. Hence, substantial cleaning is often carried out prior to any graphical analysis.

EDAmame fills this gap by offering a linear, simple, and unique dataflow, beginning with data cleaning routines. The interface allows users to hop-on and hop-off at specific stages of the workflow. In the spirit of exploratory analysis, EDAmame provides a general purpose, user-friendly interface for performing both supervised and unsupervised analysis of most modestly sized tabular data (thousands of rows with hundreds of columns). Users can guide their data through a number of correlation, clustering, and modelling protocols. Moreover, the best performing models can be visually dissected to yield information on the most important features and how they influence individual predictions.

## 2 Methods

EDAmame is an R shiny based tool that allows most tabular data to be imported and analysed. Imported data can have a combination of categorical and numerical variables, provided at least one contains target labels needed for modelling purposes. The workflow is designed to direct data through multiple transformations and visualizations to identify relationships among variables using different methods. In later steps, users can build predictive models trained on their data, assess model performance and view explanatory plots. Overall, the intuitive interface guides the user through data import, cleaning, feature distribution, dimensionality reduction, correlation and interaction analysis, predictive modelling and model explaining.

To illustrate this process, we have used a published biomedical dataset, the Wisconsin Diagnostic Breast Cancer (WDBC) data, commonly used in ML model evaluations, comprising 30 feature columns and 569 sample rows, classed as either malignant or benign (Mangasarian *et al.* 1995). Through the EDAmame tool, we will identify internal correlations within this data and, in this example, will swiftly produce high-performing predictive models comparable to reported baseline accuracy results for similar models trained on this data (Wolberg *et al.* 1992).

Within the EDAmame interface, the basic workflow is simple and laid out sequentially from left to right as individual tabs (black bullet points). It can be broadly categorized into four phases:

1. Data importation:
  - Ingest from text, clipboard, spreadsheet, URL
  - Tabulation and filtering (with type specification)
2. Graphical overview:
  - Missing data plot, optional imputation
  - Categorical feature counts
  - Numerical feature histograms
  - Dimensionality reduction (PCA, t-SNE)
3. Correlation analysis:
  - Correlation matrix
  - Paired-correlations
  - Interaction plots (two- and three-way)
4. Modelling:
  - Feature importance and selection
  - Model selection and training
  - Model performance (confusion matrix, ROC)
  - Model explanation (feature behaviour)

We now step through an example of EDAmame’s use with regards to each of these stages using the WDBC dataset.

### 2.1 Data importation

Data can be imported from most tabular formats, automatically cleaned and assigned a feature type (such as integer, float values, string, categorical and so forth). Redundant or uninformative columns (such as row numbers or lengthy descriptive columns) may also be filtered at any time using the *Filter* side menu. The final table of data to be used downstream can be inspected in the *Data* tab. For maximum flexibility, we used the datamods library (Perrier *et al.* 2024), permitting data to be imported from Excel, tab and comma-delimited files, clipboard, or online Google sheets. Once parsed, a table of detected data types is presented to the user to allow for changes if necessary (for instance, changing numeric values to strings or factors for categorical features). Rows or columns with high missingness can be filtered according to user defined cutoffs.

### 2.2 Graphical overview

**Figure 1.**
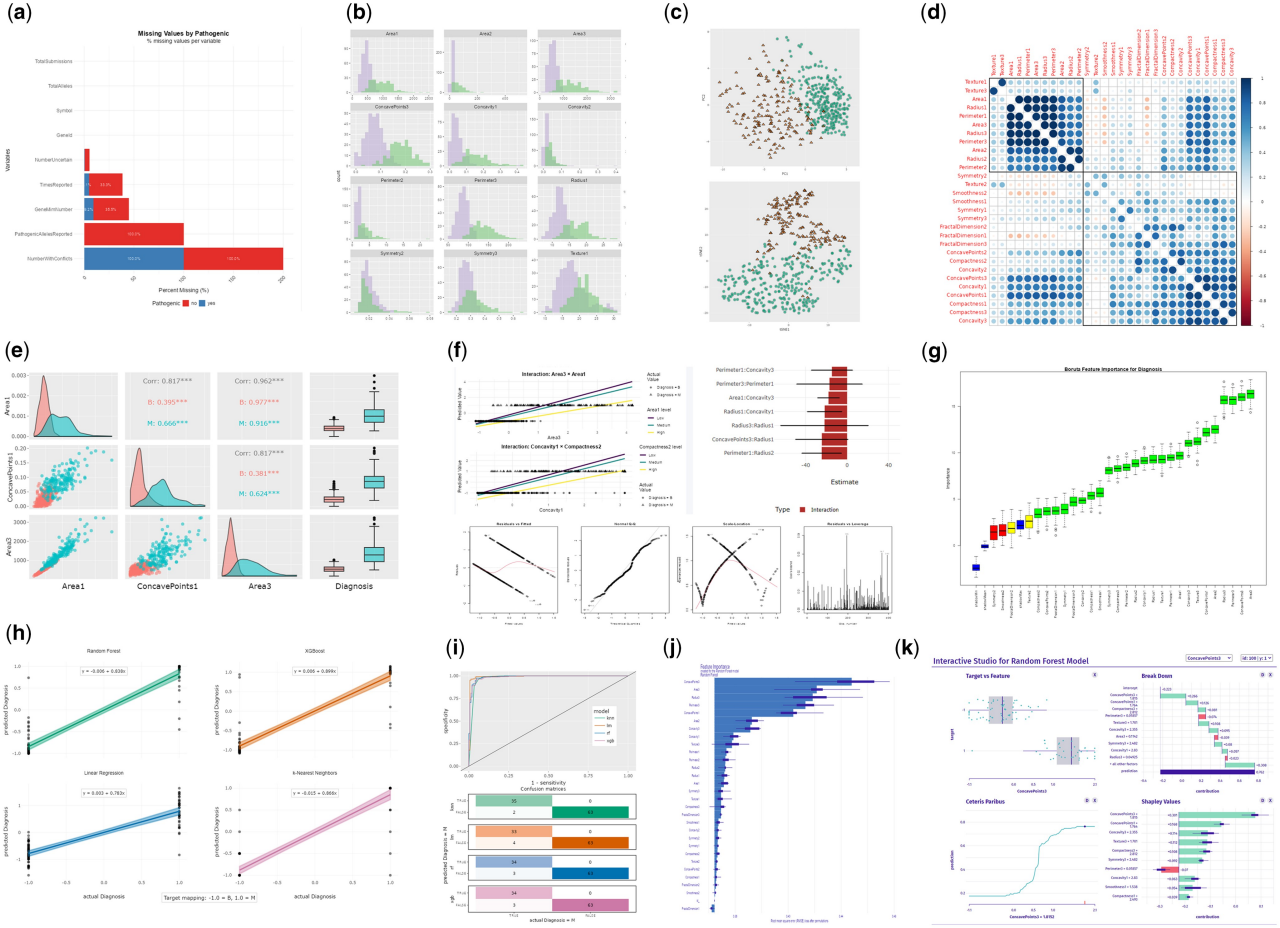
(a) missing data bar chart; (b) grouped histograms; (c) PCA (top) and t-SNE (bottom) projections; (d) correlation matrix; (e) selected paired correlations; (f) interaction plots; (g) Boruta feature importance plot; (h) linear plots of model predictions; (i) model performance ROC curves and confusion matrices; (j) feature importance plot for selected model; (k) interactive dashboard for model explaining (modelStudio).

Once imported, we can explore the WDBC data graphically. A major advantage of EDA over a series of specific tests for data quality is that EDA may reveal unexpected, hypothesis-free visual patterns that might otherwise be overlooked (Hoaglin 2003). Bar or pie charts for categorical features, and overlaid histograms for numeric features, offer clear summaries of each feature which can be grouped and coloured based on a selected label. We expected notable differences in the distributions between malignant and benign for certain features, which were indeed observed (Fig. 1b). These differences should allow ML models to reliably discriminate between these classes, which was demonstrated by early models reporting accuracies > 90% in the UCI Machine Learning repository (Mangasarian *et al.* 1995).

The problem of missing data is a pervasive issue inherent in biomedical and clinical datasets, even those from randomized controlled trials (Bell *et al.* 2014). Therefore, it is important to assess the extent of missing data in the input dataset, even after initial filtering. A stacked bar chart displays percentages of missing values for each feature. If desired, imputation can be performed using the inline tool with configurable parameters. Imputation error estimates and warnings are displayed, so it is at the discretion of the user whether to proceed with the imputed data. Since the WDBC dataset has no missing values, the Fig. 1a example is taken from a different ClinVar-based dataset (Landrum *et al.* 2016) which has features with high missingness as well as one practically absent. The user may drop additional columns or rows using the *Filter* tab in the options menu at later points in the workflow.

As a key technique of unsupervised learning, dimensionality reduction (DR) techniques are useful to identify clear outliers and clusters in the data. Principal Component Analysis (PCA) and Multidimensional Scaling (MDS) are traditional DR techniques, but they are quite sensitive to outliers (Rousseeuw and Hubert 2018). Accordingly, EDAmame offers row- and column-level filtration to enable users to clearly visualize broad patterns in their data. More recently, techniques such as t-Stochastic Neighbour Embedding (t-SNE) and Pairwise Controlled Manifold Approximation (PaCMAP) have been effectively applied to biological datasets (Huang *et al.* 2022). These offer the advantage of reducing the manifold dimensions of the data to be entirely projected into just two or three dimensions (Fig. 1c). Use of the plotly library (Plotly Technologies Inc. 2015; https://plot.ly) enables individual datapoints to be hovered over and identified, along with their other feature values for interactive visual inspection. Additionally, points can be optionally coloured, shaped and outlined according to any of their categorical features via the *Select* tab to further observe patterns of clustering.

### 2.3 Correlation analysis

In the next phase, correlation analysis can be performed in a straightforward manner and compared with the preceding DR results. Numeric features within the dataset, which are standard-normal transformed by default, are presented as a correlation matrix (Fig. 1d) in order to identify clear correlates between the features. (Large dark blue circles = high positive correlation, blanks = no correlation, and large dark red circles = high negative correlation). A more detailed inspection of subsets of features (up to eight) can be presented as informative pairwise views (Fig. 1e) of individual scatterplots, boxplots, histograms and correlation coefficients by selected category.

In the next tab, interaction modelling (Lambert *et al.* 2018) is used to identify and plot the best subset of significant two- and three-way interactions among features. This runs in under a minute on the WDBC dataset using four or more cores. However, larger datasets with many features will take longer. Accordingly, safeguards are implemented to avoid combinatorial overload. Significant (*P* < .01) two-way interactions (Fig. 1f) were found to effect diagnosis. This is supported by a Scale-Location plot, which indicates heteroscedasticity in the original data which may need to be accounted for in subsequent modelling.

### 2.4 Modelling

To logically conclude analysis of the WDBC dataset, we used EDAmame’s functionality to generate a predictive model from this data. We were then able to compare the performance of this model with that produced previously elsewhere. The tidymodels framework (Kuhn and Wickham 2020) enables the integration of multiple modelling engines to be run simultaneously through a common recipe and workflow. At the time of writing, the tidymodels website (www.tidymodels.org/packages/) lists 21 classification and regression engines with new ones added on an ongoing basis, each with differing sets of strengths and weaknesses, making it difficult to determine beforehand which might perform best on a given dataset. In this initial release, EDAmame supports nine parsnip model engines well-suited for tabular data: linear regression (including its generalized, robust, and Bayesian variants), k-nearest neighbours, radial-basis function support vector machines, random forest, extreme gradient-boosted trees, and multi-layer perceptrons (Arik and Pfister 2021).

Thereafter, EDAmame splits the data into training and test sets (80:20 split), then simultaneously builds and trains all the selected models on the target class label (Fig. 1h). Finally, 5-fold cross-validation is performed with each model to provide a comparison of model performance via the tidymodels yardstick module. The results are presented in tabular form, as well as individual receiver operating characteristic (ROC) curves and confusion matrices (Fig. 1i).

Finally, the DALEX package of XAI modules (Biecek 2018) is utilized to explain the chosen model via the *Explain* tab, which plots relative feature importance (Fig. 1j) to highlight the features contributing most to target prediction. This may assist in informing downstream experimental validation along with Boruta feature selection (Kursa and Rudnicki 2010), which is performed separately (Fig. 1g). Thanks to the common architecture of DALEX-compatible predictive models in both python and R, models built by EDAmame can also be optionally passed directly on to modelStudio (Baniecki and Biecek 2019), an external application embedded within our tool, enabling interactive exploration of various explainer plots and metrics (Fig. 1k).

## 3 Test results

An exemplary test run of the WDBC data shows that EDAmame-built models (Fig. 1i) recapitulate previous findings (Mangasarian *et al.* 1995, Wolberg *et al.* 1992) using a quick, streamlined workflow. The best performing models could clearly discriminate between malignant and benign breast cancer tumour samples in the dataset. Random forest (RF) showed the best accuracy at 99.1%, and topped most other metrics including sensitivity, recall, F1 score, kappa and Matthews Correlation Coefficient (MCC). Traditional linear modelling, though slightly lower in accuracy (98.3%), outperformed RF at area under ROC (0.991) and Precision-Recall (0.989) curves. These compare well with the baseline model performance figures reported at the UC Irvine Machine Learning Repository: 97.9% median accuracy for RF, and 95.8% for logistic regression (Wolberg *et al.* 1992).

## 4 Conclusion

With EDAmame, we present a graphical application for exploratory data analysis that uses a simplified workflow for highlighting associations and performing basic modelling to obtain valuable insights from tabular datasets. Use of the tool requires no programming experience or command-line interaction. Analysis can provide information on overall data quality and differentiability on selected labels, while preliminary hypotheses can be tested using a suite of classification models. The best performing models can be further explained to uncover the most important features for prediction, all in an intuitive, user-friendly interface.

## Data Availability

No new data were generated in support of this release. Cited example datasets can be accessed from https://edamame.org.au/ or https://doi.org/10.5281/zenodo.15356492.
